# Pericapsular Nerve Group (PENG) Block for Pediatric Hip Surgery: A Report of Three Cases

**DOI:** 10.7759/cureus.78996

**Published:** 2025-02-14

**Authors:** Amanda M Bunnell, Jennifer V Smith

**Affiliations:** 1 Anesthesiology and Perioperative Medicine, Medical University of South Carolina, Charleston, USA

**Keywords:** hip surgery, pediatric anesthesia, pediatric hip pain, pediatric hip surgery, pediatric regional anesthesia, pericapsular nerve group block, regional anesthesia and chronic pain

## Abstract

Pain control for pediatric patients undergoing hip surgery can be challenging. These patients are often medically and/or surgically complex and may have numerous comorbidities. Using a multimodal strategy to ensure adequate analgesia for these patients should include regional techniques when possible. However, historical techniques for regional analgesia may be contraindicated in medically complex children, provide inadequate coverage of the region, or cause undesired weakness. Pericapsular nerve group block is a relatively novel technique, better described in the adult literature, which targets the specific sensory nerves innervating the anterior capsule of the hip. It can be combined with the lateral femoral cutaneous block for cutaneous analgesia of the hip region. In this case report, we demonstrate the feasibility of this combined technique in three pediatric patients who underwent hip surgery.

## Introduction

Historical techniques for analgesia of the hip have consisted of fascia iliaca, quadratus lumborum, lumbar plexus, and femoral nerve blocks, with imperfect efficacy. The pericapsular nerve group (PENG) block was described by Giron-Arango et al. in 2018 as a block of the articular nerve branches of the hip [1]. The PENG block targets accessory branches of the obturator, accessory obturator, and femoral nerves, aiming to provide complete sensory analgesia of the anterior hip while sparing the motor branches. In adult populations, the PENG block is commonly paired with the lateral femoral cutaneous block (LFCN) for cutaneous analgesia of the hip. The combination of PENG and LFCN blocks has gained significant popularity in the adult hip surgery population, particularly for its motor-sparing effect. Motor weakness is one of the major disadvantages of traditional techniques such as femoral, lumbar plexus, fascia iliaca, and epidural blocks, as it impairs patients from participating in early physical therapy. While there is some debate in the adult literature over the efficacy of the PENG block for hip fracture, a recent meta-analysis concluded that the PENG block provides better analgesia in the first 24 hours after hip arthroplasty, though questioned the clinical relevance given high heterogeneity across studies [2].

Use of the PENG-LFCN in pediatrics has been limited despite a large pediatric population of patients with developmental dysplasia of the hip. Many of these patients have neuromuscular disease and are medically complex with comorbidities including cardiopulmonary disease [3], in whom a regional technique is ideal to limit respiratory depression associated with opioids.

At the time of our review and to the best of our knowledge, current pediatric literature is limited to several international case reports [4-9] and one report on a PENG catheter [10]. Only two include a combined block of the LFCN [4,8]. We present three cases of PENG and LFCN blocks in pediatric patients for hip surgery. Consent and assent, where appropriate, were obtained for disclosure of patient health information with a Health Insurance Portability and Accountability Act (HIPAA) Authorization Form obtained. This manuscript adheres to the applicable EQUATOR guideline (https://www.equator-network.org) and the CARE statement (CARE Case Report Guidelines).

## Case presentation

Case 1

A 14-year-old male, weighing 39.2 kg, with a left femoral neck fracture secondary to an all-terrain vehicle (ATV) accident, status-post open reduction and internal fixation, complicated by avascular necrosis, displacement and loss of fracture fixation, status-post hardware removal presents for left total hip arthroplasty. After a discussion of risks and benefits with the patient and his family, the decision was made to proceed with a spinal anesthetic with PENG and LFCN blocks for post-operative pain, as per our typical institutional approach to adult hip arthroplasty, avoiding techniques that would cause muscle weakness so that our patient could participate in early physical therapy. The patient was pre-medicated with 2 mg of midazolam intravenously (IV). Upon arrival to the operating room, the patient was placed in the sitting position, further sedated with propofol 20 mg IV and dexmedetomidine 8 mcg IV, and a subarachnoid block was placed with 12.5 mg (0.3 mg/kg) of isobaric bupivacaine at the L4-5 interspace. The patient was returned to the supine position for peripheral nerve block placement.

**Figure 1:**
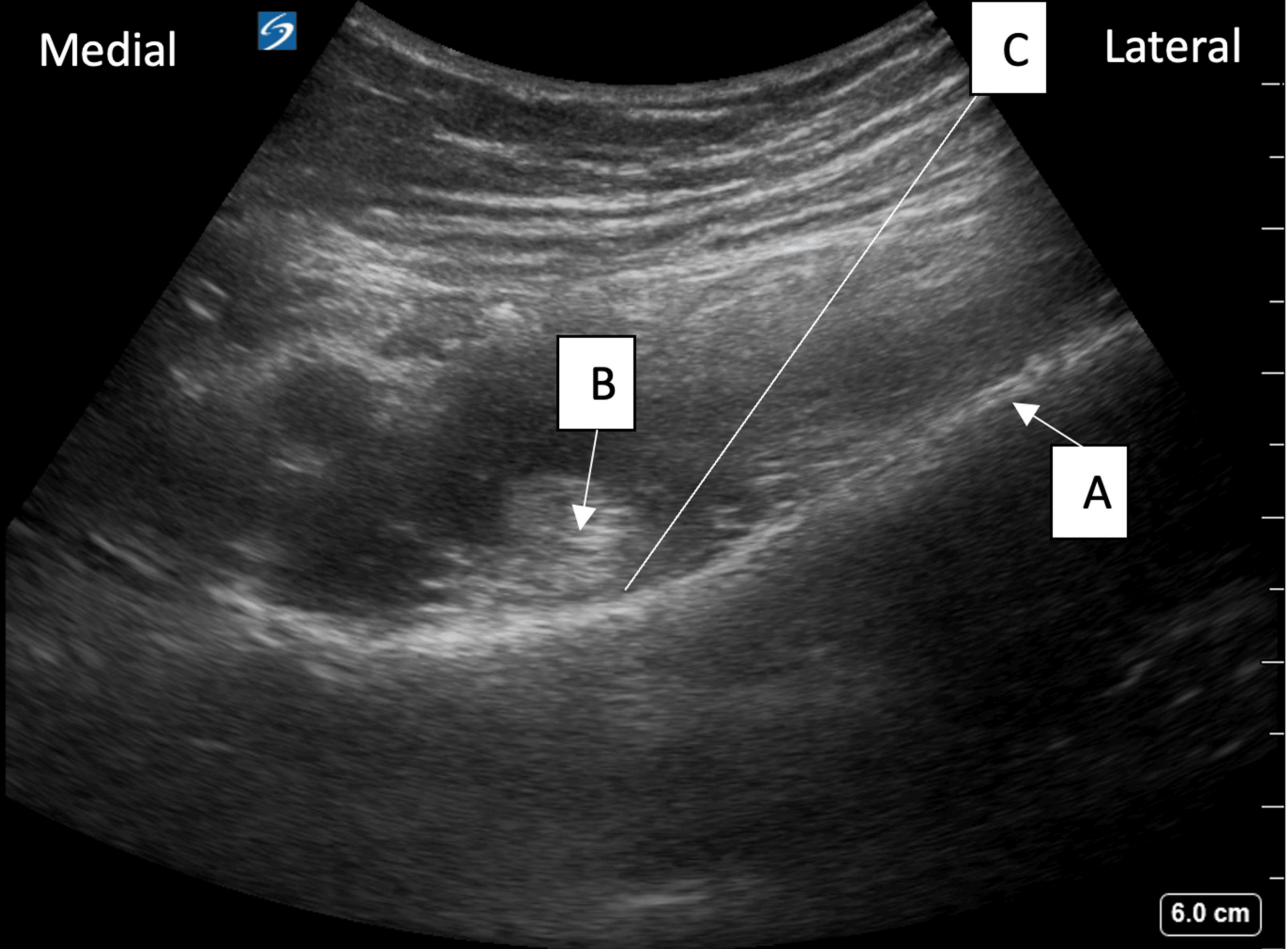
An example of PENG ultrasound anatomy The image shows the anterior inferior iliac spine (A), psoas tendon (B), and trajectory of the needle (C). PENG: Pericapsular nerve group.

The left-sided PENG block was performed with an 8-3 MHz curvilinear ultrasound transducer placed in the transverse orientation at the anterior superior iliac spine, moving inferiorly to visualize the anterior inferior iliac spine and then rotating approximately 45 degrees to visualize the iliopubic eminence and the psoas tendon (Figure 1).

With an in-plane technique from lateral to medial, 15 ml of 0.2% bupivacaine (0.4 ml/kg) was injected following negative aspiration to the fascial plane between the psoas tendon and the ilium (21-gauge, 90 mm, StimuQuik Echo Insulated Peripheral Nerve Block Needle; Teleflex®, Reading PA, USA).

**Figure 2:**
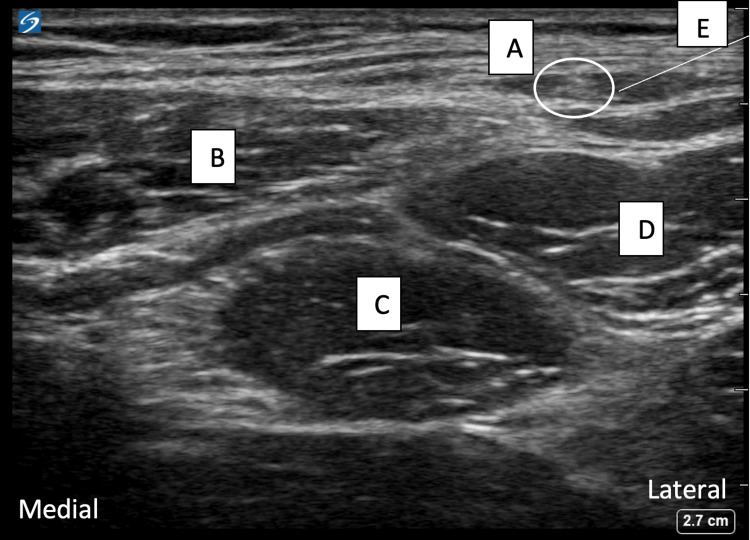
An example of LFCN ultrasound anatomy The image shows the lateral femoral cutaneous nerve (A), sartorius muscle (B), rectus femoris (C), tensor fascia lata (D), and needle trajectory (E). LFCN: Lateral femoral cutaneous block.

The left-sided LFCN block was performed utilizing a 13-6MHz linear ultrasound transducer placed inferior to the anterior superior iliac spine to visualize the sartorius and tensor fasciae latae muscles. The LFCN is seen bordering the superolateral aspect of the sartorius muscle (Figure 2).

The needle was inserted in-plane from lateral to medial and 5 ml of 0.2% ropivacaine (approximately 0.1 ml/kg) was injected after negative aspiration around the LFCN. The total PENG-LFCN local anesthetic dose was 1 mg/kg. After confirmation of an adequate sensory level for the subarachnoid block, the patient was maintained on a propofol infusion for sedation, and the procedure proceeded uneventfully. Aside from fentanyl 10 mcg IV during block placement, the remainder of the anesthetic was opioid-free. Additional intra-operative medications included dexamethasone 4 mg IV bolus for post-operative nausea and vomiting prophylaxis at the beginning of the procedure and dexmedetomidine 12 mcg IV in split boluses at the end of the procedure. The surgeon performed pericapsular irrigation during the procedure with 8 ml 0.5% ropivacaine (1 mg/kg) mixed with epinephrine 0.5 mg and clonidine 80 mcg as per his typical arthroplasty protocol. At the conclusion of the procedure, the patient was transported to the post-anesthesia care unit, where he reported no pain during his stay.

During the first 24 hours post-operatively, his pain was managed with acetaminophen and oxycodone as needed. He required four doses of oxycodone 5 mg per os (PO), the first at nine hours post-procedure or 12 hours after block placement (Table 1).

**Table 1:** Objective data for each case yo: Years old; M: Male; F: Female; PENG: Pericapsular nerve group; LFCN: Lateral femoral cutaneous block; IV: Intravenous; PO: Per OS.

Case	Patient data	Primary Anesthetic	Block Dose	Intra-operative Opioid Analgesic Medication	Intra-operative Non-opioid Medication	PACU Opioid Medications	Floor Opioid Medications	Time from Block Placement to First Post-Operative Opioid (hh:mm)
1	14yo M 39.2 kg	Spinal (bupivicaine 0.5% 2.5 ml with fentanyl 15 mcg)	PENG: ropivacaine 0.2% 0.4 ml/kg + LFCN: ropivacaine 0.2% 0.1 ml/kg	None	Midazolam IV 2 mg x 1 dose; dexamethasone IV 4mg x 1 dose; dexmedetomidine IV 0.5 mcg/kg in divided doses	None	Oxycodone PO 5 mg x 4 doses	12:13
2	3yo F 16.4 kg	General Endotracheal	PENG: ropivacaine 0.2% 0.6 ml/kg + LFCN: Ropivacaine 0.2% 0.3 ml/kg	Fentanyl IV 1.5 mcg/kg in divided doses	Acetaminophen PO 15 mg/kg (Pre-op); ketorolac IV 0.5 mg/kg; dexamethasone IV 0.5 mg/kg; dexmedetomidine IV 0.75 mcg/kg in divided doses	None	None	N/A
3	2yo F 15.2 kg	General Endotracheal	PENG: ropivacaine 0.2% 0.4 ml/kg + LFCN: ropivacaine 0.2% 0.2 ml/kg	Fentanyl 2.3 mcg/kg in divided doses	Acetaminophen IV 15 mg/kg x 1 dose; dexamethasone IV 0.1 mg/kg x 1 dose; ketamine IV 0.3 mg/kg x 3 doses; ketorolac IV 0.5 mg/kg x 1 dose	None	Morphine IV 0.05 mg/kg x 1 dose	17:26

Pain scores ranged from 0-5 (Table 2).

**Table 2:** Pain scores for each patient pre-operatively, in PACU, and post-operatively on the floor FLACC: Face, Legs, Activity, Cry, and Consolability Scale; NR: Not recorded; PACU: Post anesthesia care unit.

Case	Scale	Pre-operative	PACU	One Hour	Eight Hours	Nine Hours	13 Hours	14 Hours	19 Hours	21 Hours	23 Hours	28 Hours
1	Numeric	0	0	0	NR	3	NR	NR	2	3	5	3
2	FLACC	0	0	0	NR	0	NR	NR	NR	0	NR	NR
3	FLACC	NR	NR	0	8	NR	10	0	NR	0	NR	NR

He participated in physical therapy on post-operative days 0-1 and was discharged home on post-operative day 1. The patient and his family were satisfied with his pain control. There were no noted complications on his post-operative follow-ups.

Case 2

A three-year-old, 16.4 kg female with developmental hip dysplasia and no other pertinent medical history presented for unilateral hip osteotomy and spica cast placement. After inhalational mask induction and peripheral intravenous line placement, the patient was endotracheally intubated and unilateral PENG and LFCN blocks were placed in a similar fashion to case 1, except for the use of a 13-6MHz linear ultrasound transducer used in place of the curvilinear transducer given the patient’s smaller size. Ropivacaine 0.2% was used for both PENG and LFCN blocks, dosed at 0.6 ml/kg and 0.3 ml/kg, respectively.

The surgery proceeded uneventfully. She was extubated and brought to the recovery room in stable condition. The patient received fentanyl 1.5 mcg/kg intra-operatively, of which 1 mcg/kg was given just prior to endotracheal intubation. Additional intra-operative medications included ketorolac IV 0.5 mg/kg, dexamethasone IV 0.5 mg/kg, dexmedetomidine IV 0.75 mcg/kg, and acetaminophen PO 15 mg/kg (given pre-operatively). Post-operatively, she did not receive any opioid medication and analgesia was managed satisfactorily with scheduled acetaminophen per os (PO) and ibuprofen PO. The patient’s caregivers were satisfied with her pain control. She was discharged on post-operative day 1. There were no noted complications at surgical follow-up appointments.

Case 3

A two-year-old, 15.2 kg female with developmental delay, right hip dysplasia, and right hip dislocation presented to the operating room for open reduction and internal fixation of her right hip and spica cast placement. The patient was pre-medicated with midazolam 0.5 mg/kg PO and underwent mask induction. Intravenous access was obtained, and the patient was subsequently intubated. Right-sided PENG and LFCN blocks were placed as above, with ropivacaine 0.2%, dosed 0.4 ml/kg and 0.2 ml/kg, respectively. Additional intra-operative medications included fentanyl 2.3 mcg/kg IV in divided doses, acetaminophen 15 mg/kg IV, dexamethasone 0.1 mg/kg IV, and ketamine 0.3 mg/kg IV in divided doses. The surgery was completed uneventfully, and the patient was comfortable in the recovery room, requiring no additional pain medications. During the first 24 hours post-operatively, she received one dose of morphine 0.05 mg/kg IV at 13 hours post-procedure and was otherwise comfortable on her regimen of scheduled oral acetaminophen and ibuprofen. She was discharged home on post-operative day one. Her caregivers were satisfied with her pain control and there were no noted complications.

## Discussion

The PENG block is a relatively novel regional anesthesia technique aimed at providing analgesia to the hip and has been viewed as an alternative to other techniques such as epidural, lumbar plexus, quadratus lumborum, fascia iliaca, or femoral nerve blockade. These traditional techniques that lack efficacy due to the complex innervation of the hip joint cause weakness impairing participation in physical therapy, or can be a higher risk for other complications such as hypotension, bleeding, and damage to surrounding structures, as in the case of the neuraxial and lumbar plexus techniques. Neuraxial techniques may also be contraindicated due to patient comorbidities such as spinal cord defects or coagulopathy. While the PENG block has been studied rather extensively in the adult population for surgery such as total hip arthroplasty, pediatric literature is limited to a few case reports and no prospective analysis.

Furthermore, there is a significant population of pediatric patients with neuromuscular disease who present for hip and pelvic procedures, for which an optimal regional technique is lacking. These patients are often poor candidates for caudal or epidural placement; they may be at higher risk for respiratory complications compounded by narcotic use, and blocks such as the quadratus lumborum lack comprehensive coverage of the hip joint.

Orozco et al. reported the first use of a PENG block in a pediatric patient, an eight-year-old, for the removal of osteosynthetic material s/p repair of a femur fracture [4]. While this case did combine the use of PENG and LFCN blocks, a femoral block was also used for analgesia, making it challenging to tease out which block was most efficacious for this patient.

Subsequent case reports described the use of PENG blocks in pediatric patients without combination with the LFCN block [5,9]. Acharya and Lamsal reported on the use of an isolated PENG block to provide analgesia in a 16-year-old patient with a hip fracture to facilitate the sitting position for placement of a neuraxial block but did not comment on post-operative pain [6]. Ince et al. reported the use of a PENG block combined with an erector spinae plane block for a four-year-old undergoing a derotational osteotomy for a congenitally dysplastic hip, providing the patient with seemingly successful analgesia but again clouding the picture with multiple blocks [7].

In the adult population, a case series by Roy et al. described PENG blocks on five patients undergoing hip surgery [11]. The authors noted that some of their patients complained of pain in the dermatomal distribution of the lateral femoral cutaneous nerve, requiring rescue analgesia. They then performed combination PENG and LFCN blocks on an additional five patients for hip surgery and noted that none required rescue analgesia, suggesting that PENG and LFCN in combination provide complete analgesia for hip surgery.

To the best of our review, there has only been one international case report in pediatrics on the combined PENG and LFCN technique for pediatric hip surgery [8]. Our three cases of PENG block in combination with LFCN suggest that the combined technique is feasible and has a good analgesic effect for our patients. While patient number 2 did have some elevated FLACC (Face, Legs, Activity, Cry, Consolability) scale scores overnight, she was otherwise comfortable and only required one dose of opioid medication, after which she remained comfortable.

One limitation of the combination of LFCN and PENG blocks for the pediatric population is patient size limits the volume of local anesthetic, particularly if the surgery is bilateral and thus necessitates four peripheral nerve blocks. Based on our experience, we suggest a minimum patient size of 12 kg for unilateral and 24 kg for bilateral PENG blocks in combination with LFCN. If utilizing ropivacaine 0.2%, this would allow for a dose of 0.3-0.35 ml/kg/side (max 20 ml) for the PENG block and 0.15-0.2 ml/kg/side (max 10 ml) for the LFCN block. However, optimal volume and concentration of local anesthetic remains a topic for further exploration.

While risks of the block include vascular puncture, bleeding, and damage to surrounding structures such as the nearby femoral or lateral femoral cutaneous nerves, to date, there have been no major adverse events reported in the literature. We argue that the PENG block, in combination with the LFCN, offers more comprehensive analgesia of the hip joint than other previous techniques for pediatric hip surgery. Further prospective research is necessary to demonstrate its safety and efficacy versus other techniques.

## Conclusions

Achieving adequate analgesia for pediatric patients undergoing hip surgery can be challenging. Although traditional neuraxial techniques such as caudal and epidural may provide effective analgesia, they may be contraindicated in complex patients with spinal cord anomalies or coagulopathies. Peripheral nerve block techniques such as the quadratus lumborum may not provide adequate analgesia due to complex innervation of the joint, while the lumbar plexus block may have an unfavorable risk profile. This report suggests the feasibility of the PENG and LFCN block techniques for analgesia for pediatric patients undergoing hip surgery. While our observations are promising, further prospective studies are necessary to establish both the safety and efficacy of this approach in children undergoing hip surgery.
